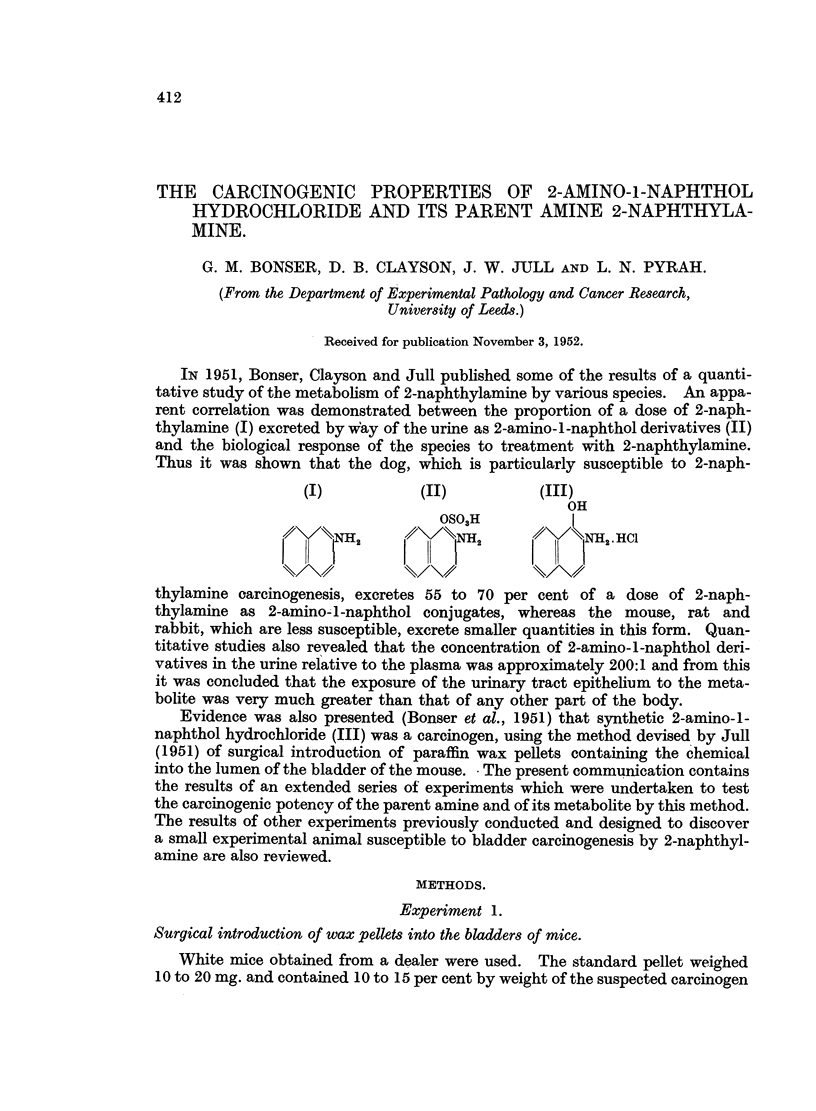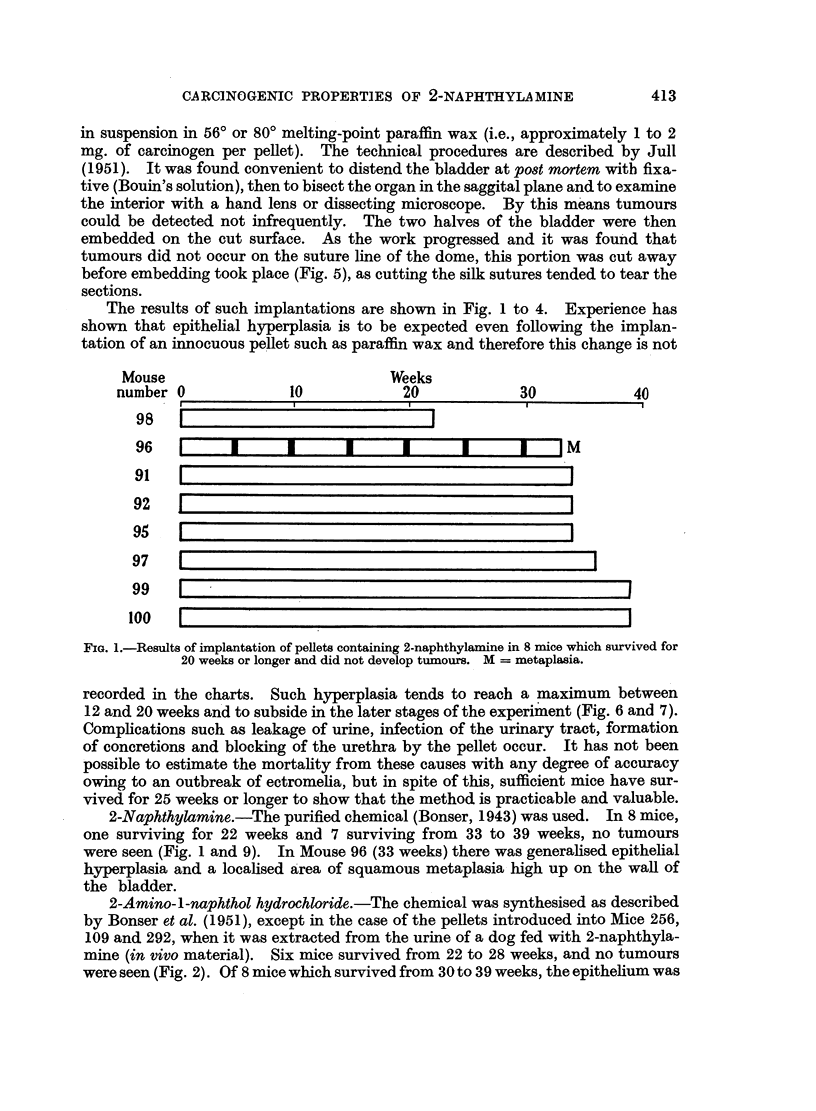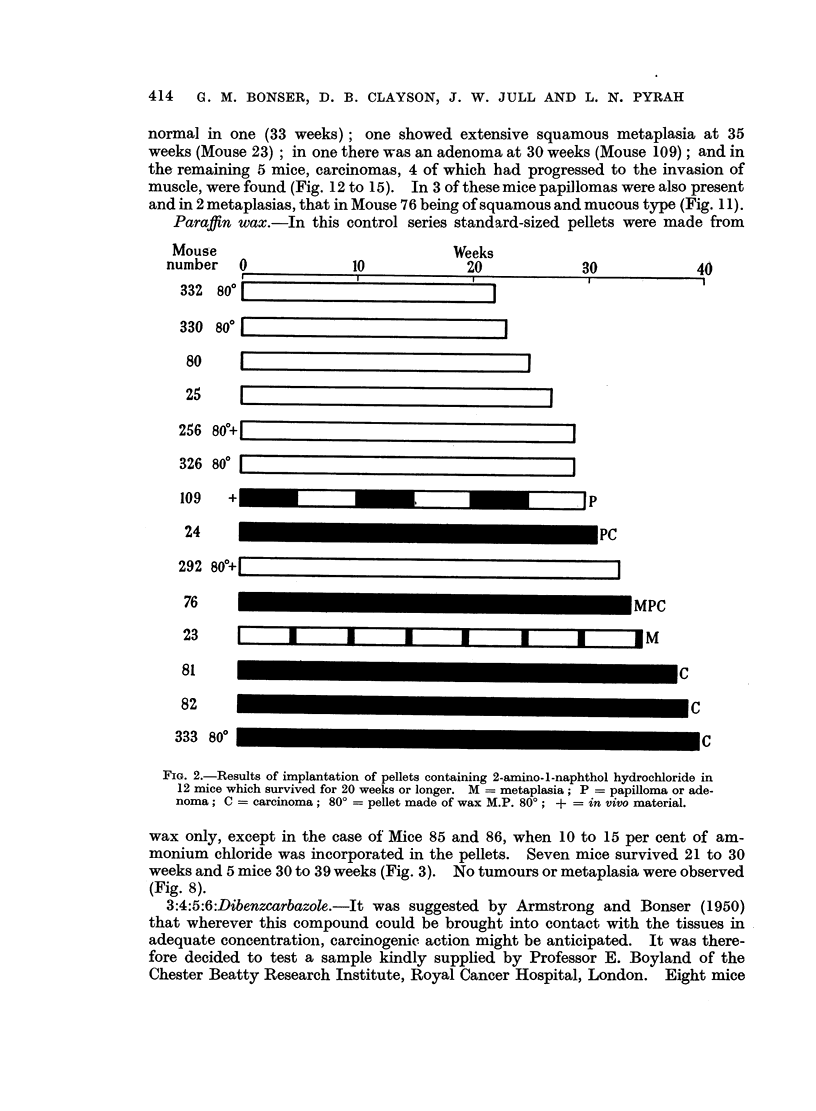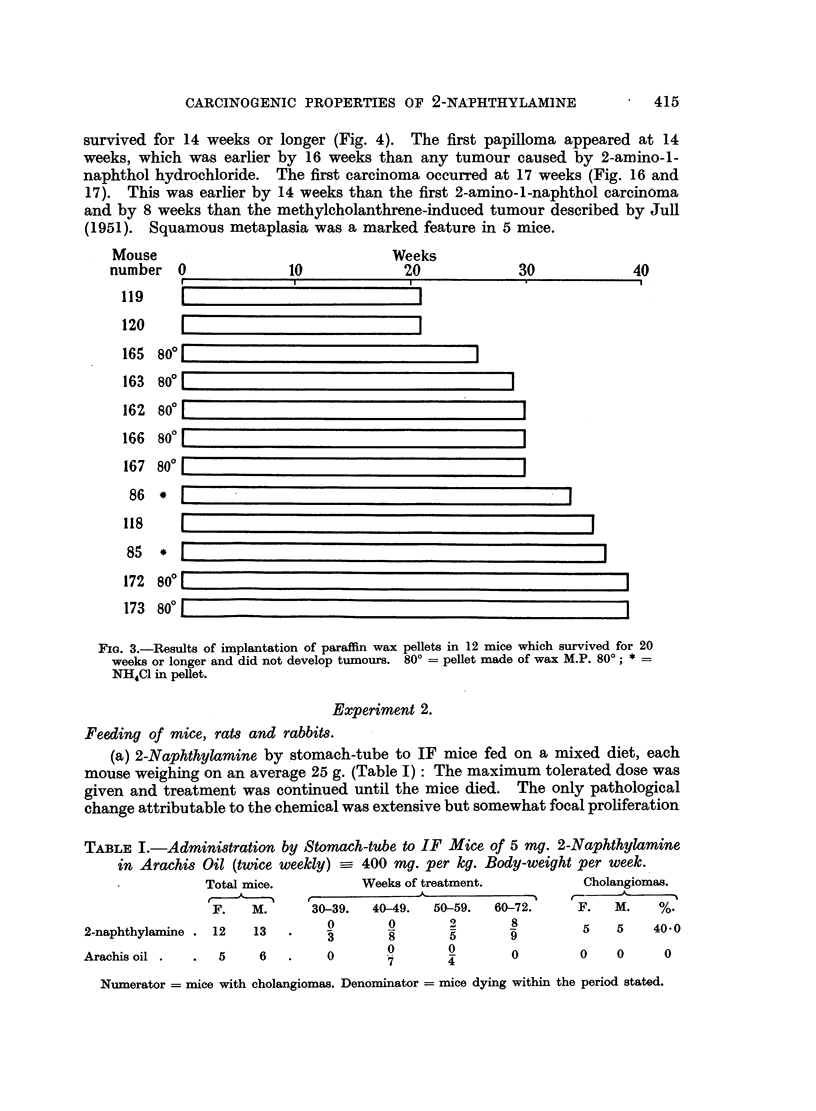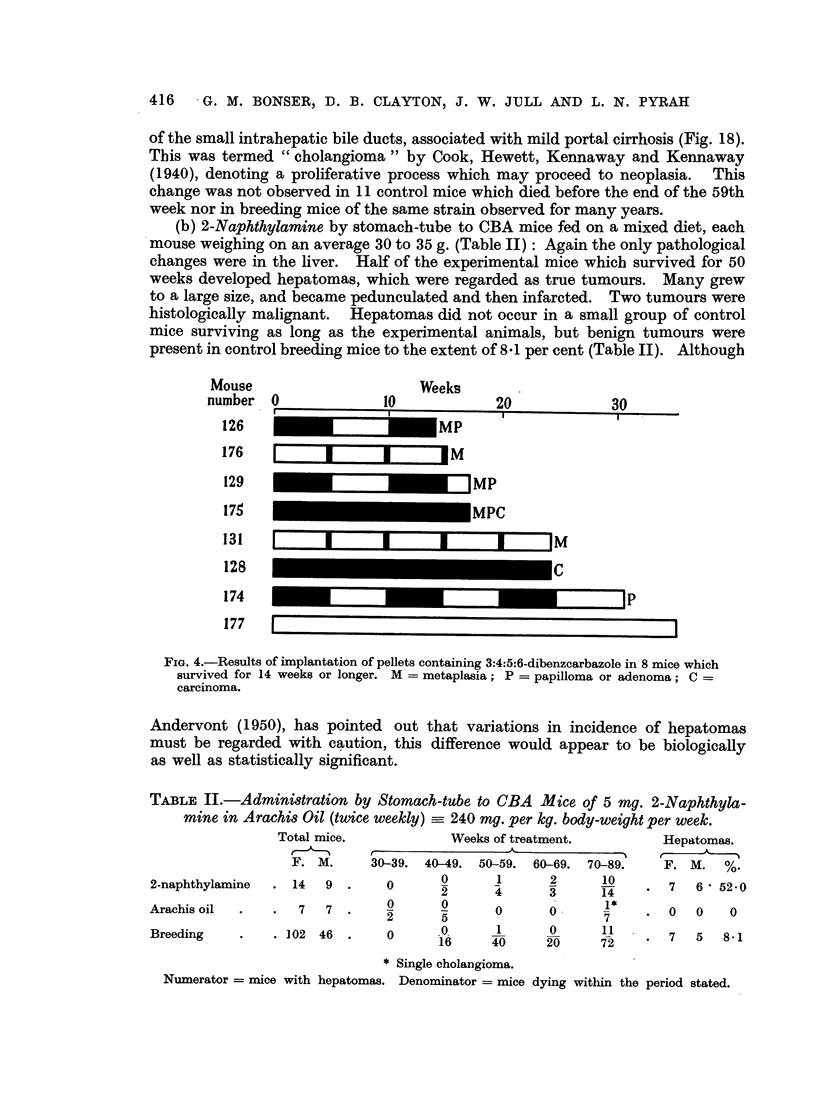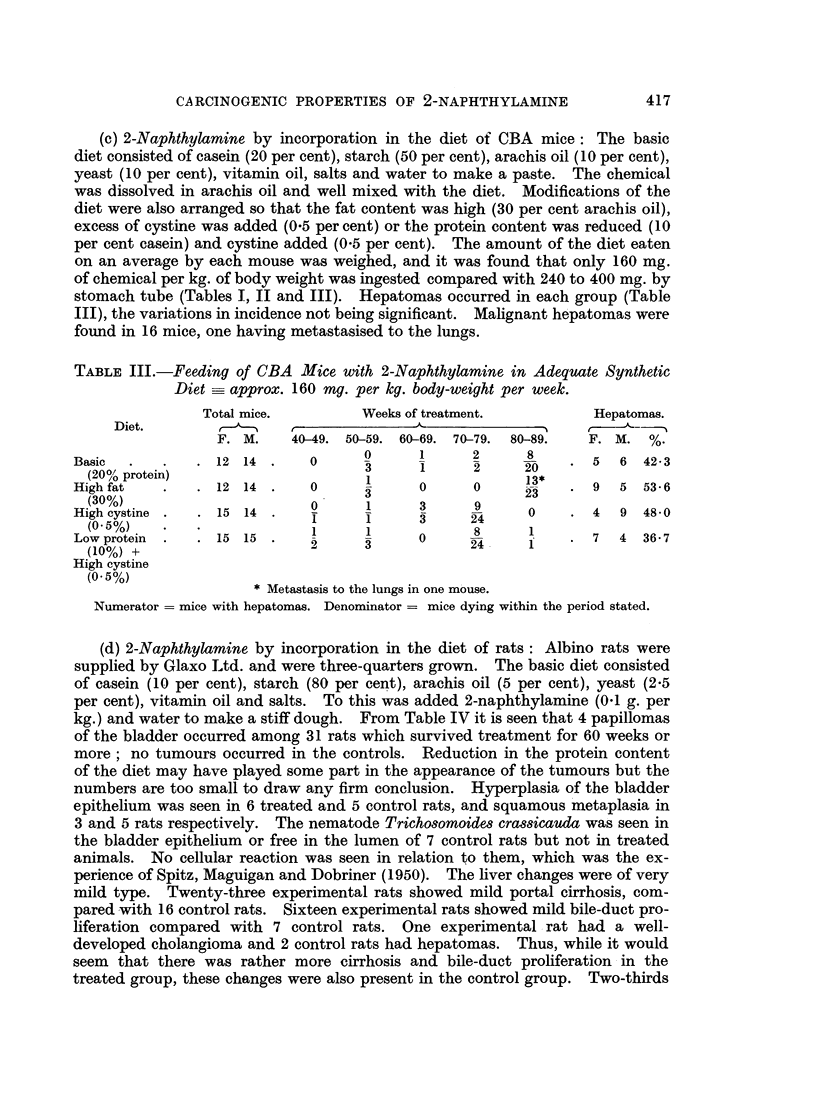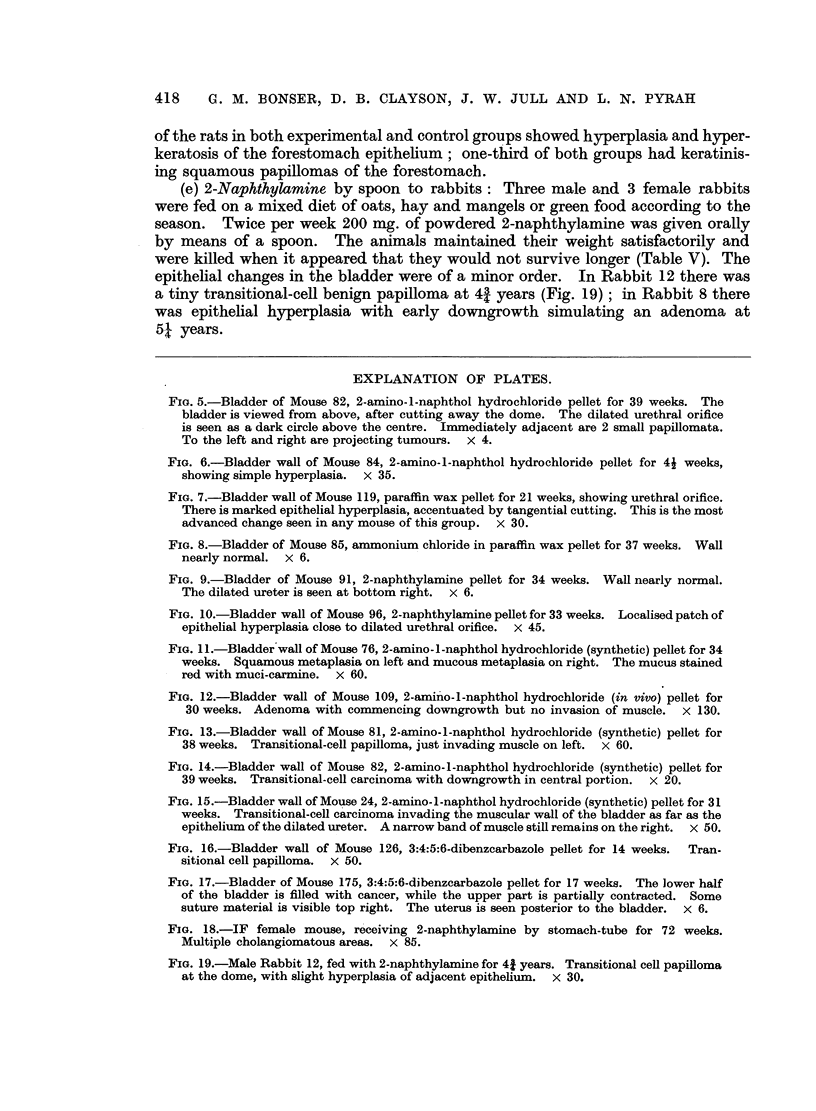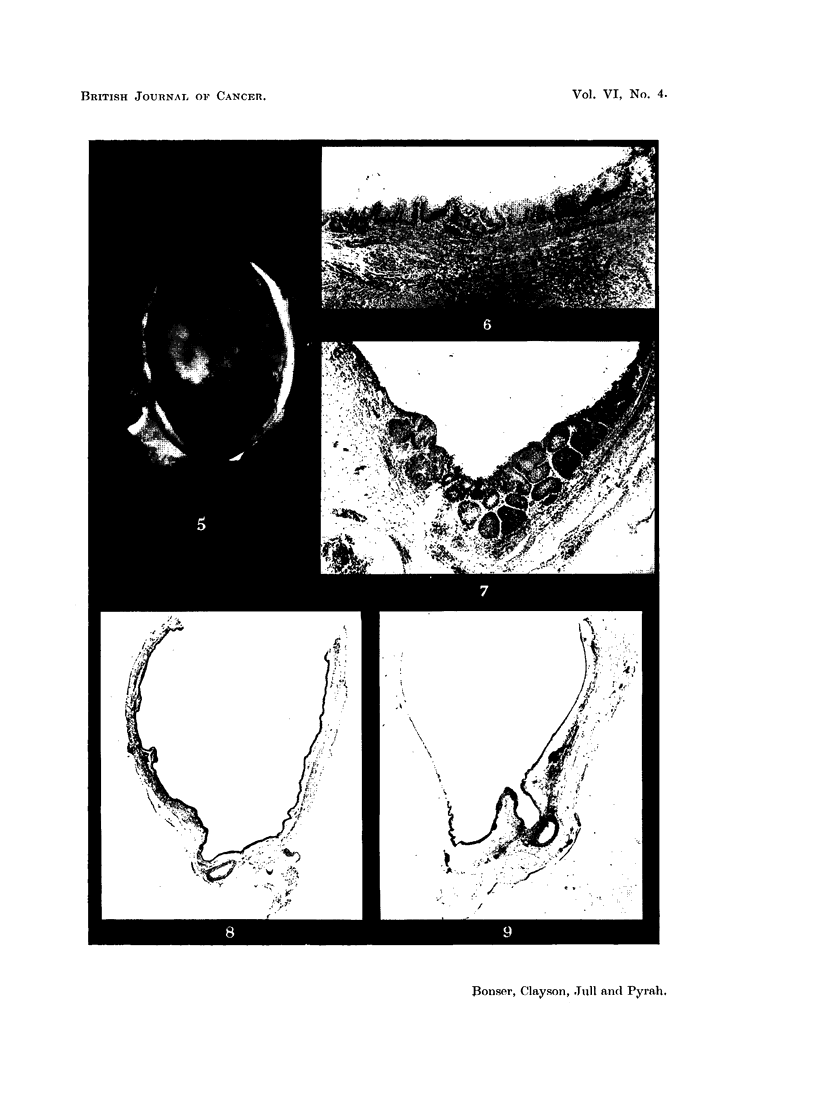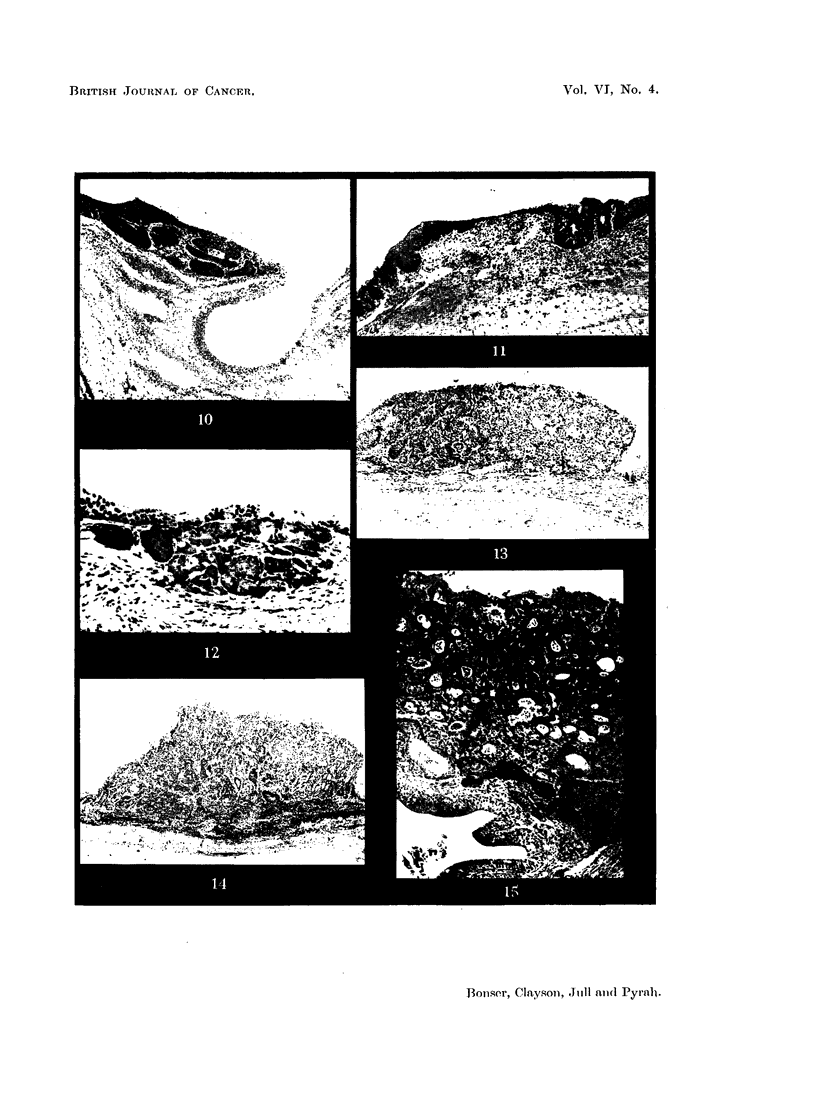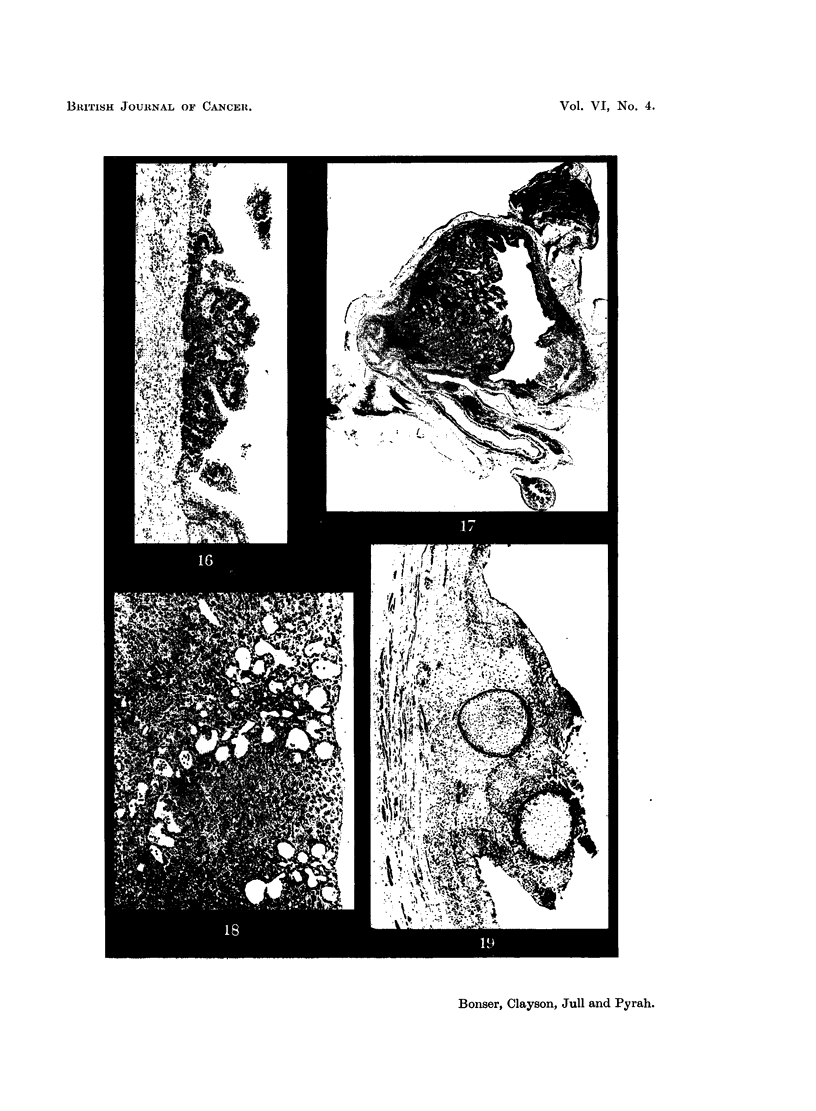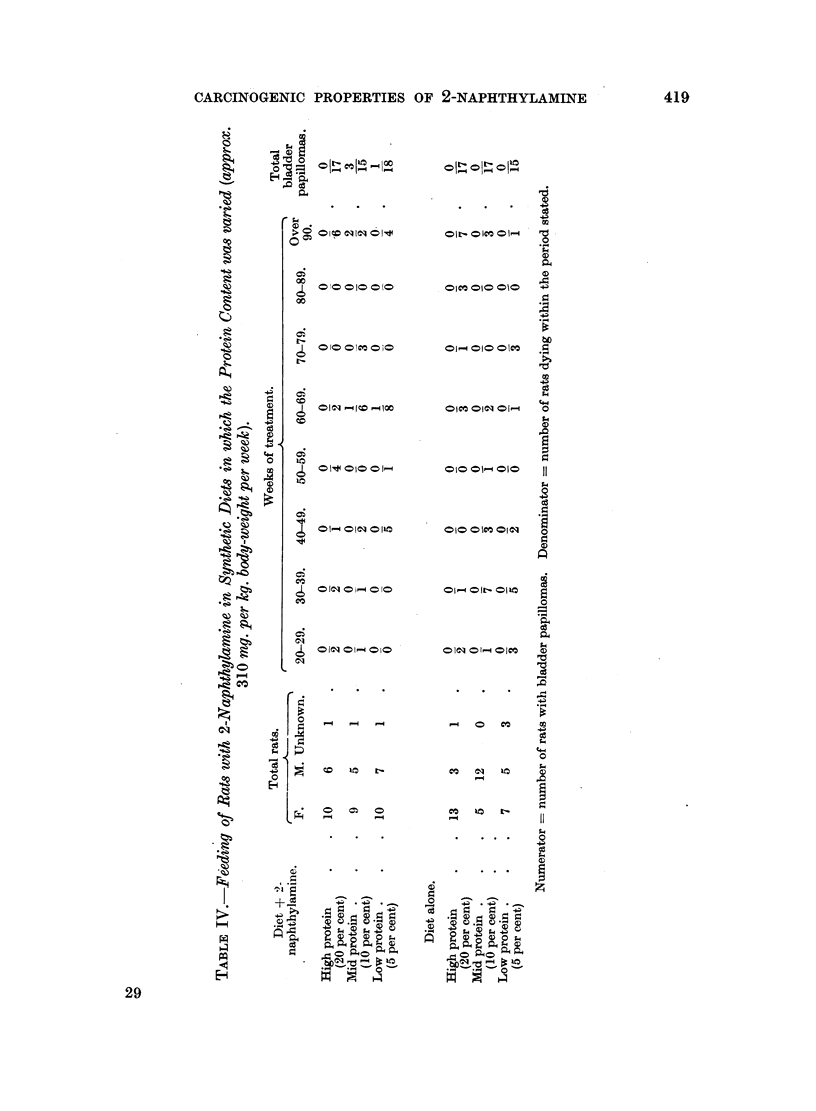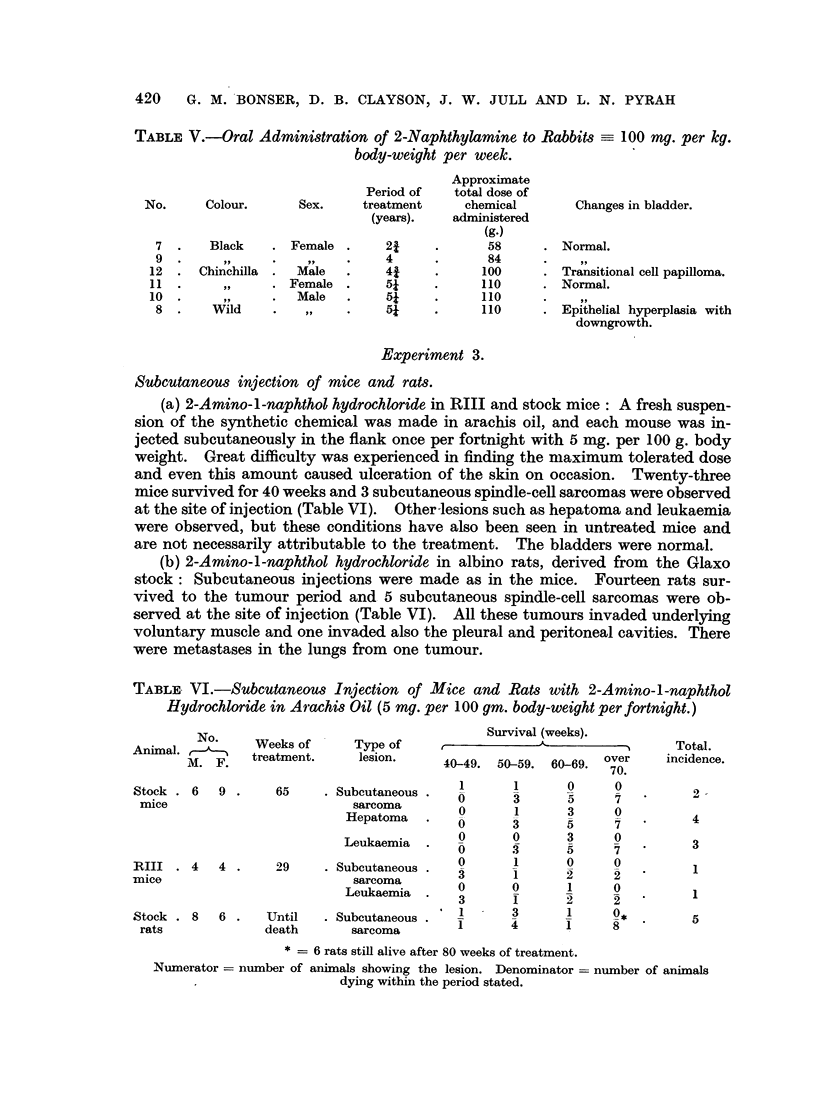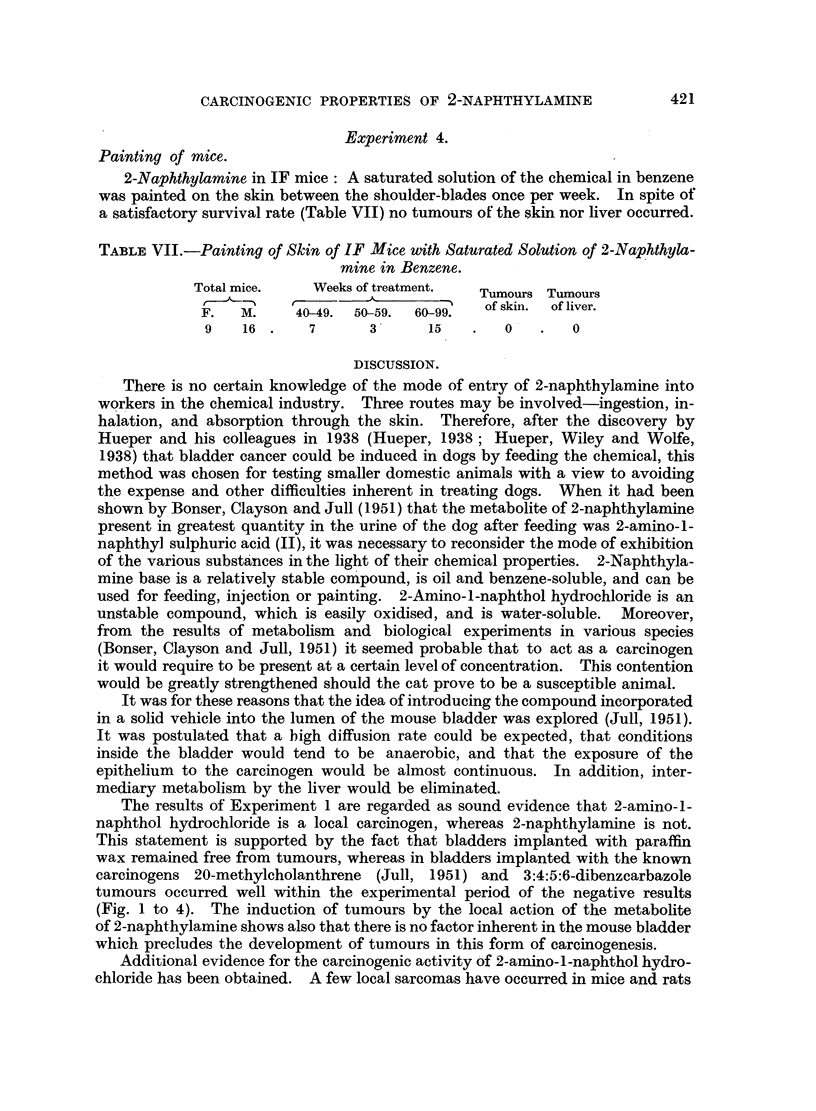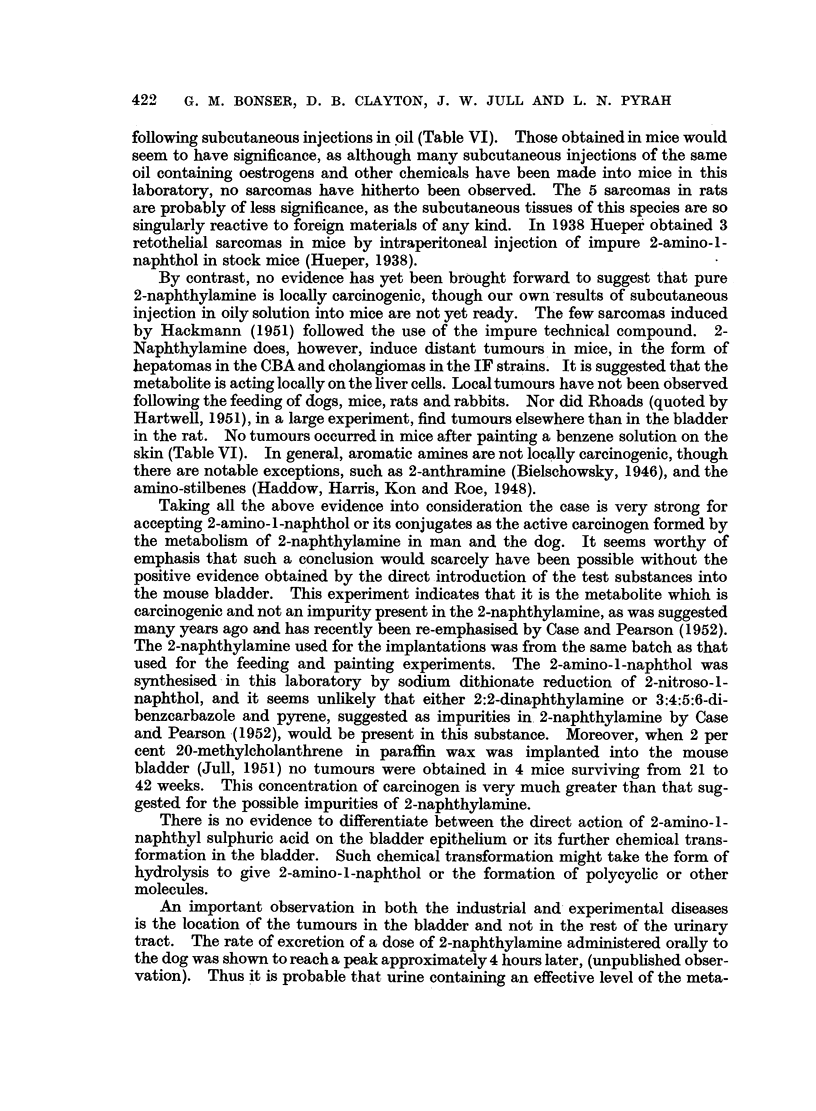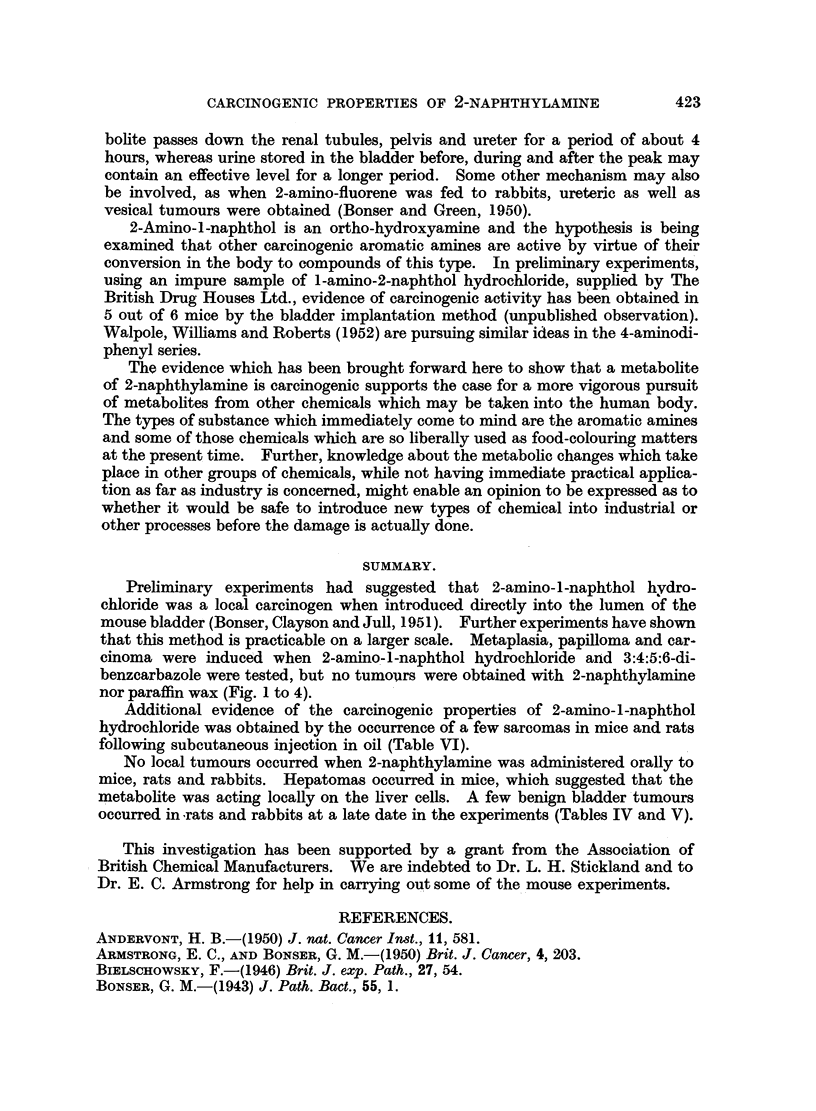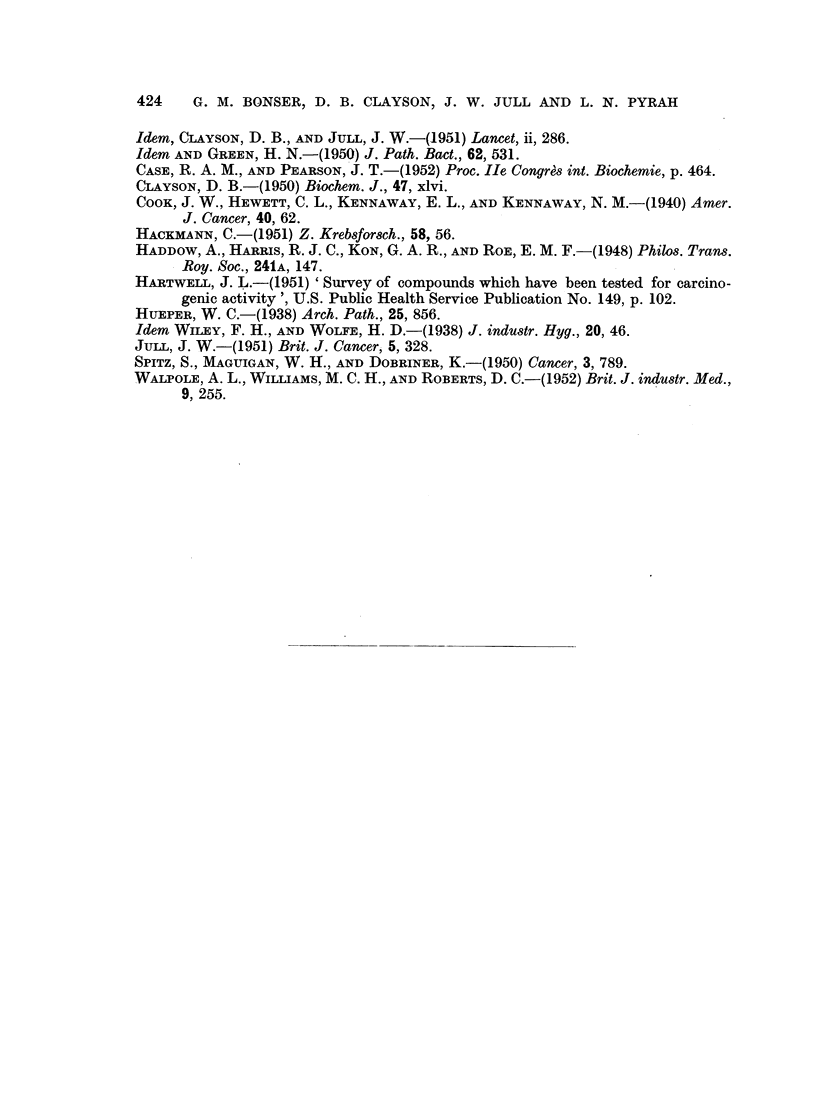# The Carcinogenic Properties of 2-Amino-1-Naphthol Hydrochloride and its Parent Amine 2-Naphthylamine

**DOI:** 10.1038/bjc.1952.47

**Published:** 1952-12

**Authors:** G. M. Bonser, D. B. Clayson, J. W. Jull, L. N. Pyrah

## Abstract

**Images:**


### THE CARCINOGENIC PROPERTIES OF 2-AMINO-1-NAPHTHOL HYDROCHLORIDE AND ITS PARENT AMINE 2-NAPHTHYLAMINE.

G. M. BONSER, D. B. CLAYSON, J. W. JULL AND L. N. PYRAH.

(*From the Department of Experimental Pathology and Cancer Research, University of Leeds.*)

Received for publication November 3, 1952.

IN 1951, Bonser, Clayson and Jull published some of the results of a quantitative study of the metabolism of 2-naphthylamine by various species. An apparent correlation was demonstrated between the proportion of a dose of 2-naphthylamine (I) excreted by way of the urine as 2-amino-1-naphthol derivatives (II) and the biological response of the species to treatment with 2-naphthylamine. Thus it was shown that the dog, which is particularly susceptible to 2-naph-

(I)

(II)

(III)

thylamine carcinogenesis, excretes 55 to 70 per cent of a dose of 2-naphthylamine as 2-amino-1-naphthol conjugates, whereas the mouse, rat and rabbit, which are less susceptible, excrete smaller quantities in this form. Quantitative studies also revealed that the concentration of 2-amino-1-naphthol derivatives in the urine relative to the plasma was approximately 200:1 and from this it was concluded that the exposure of the urinary tract epithelium to the metabolite was very much greater than that of any other part of the body.

Evidence was also presented (Bonser *et al.*, 1951) that synthetic 2-amino-1-naphthol hydrochloride (III) was a carcinogen, using the method devised by Jull (1951) of surgical introduction of paraffin wax pellets containing the chemical into the lumen of the bladder of the mouse. The present communication contains the results of an extended series of experiments which were undertaken to test the carcinogenic potency of the parent amine and of its metabolite by this method. The results of other experiments previously conducted and designed to discover a small experimental animal susceptible to bladder carcinogenesis by 2-naphthylamine are also reviewed.

### METHODS.

### *Experiment* 1.

*Surgical introduction of wax pellets into the bladders of mice.*

White mice obtained from a dealer were used. The standard pellet weighed 10 to 20 mg. and contained 10 to 15 per cent by weight of the suspected carcinogen in suspension in 56° or 80° melting-point paraffin wax (i.e., approximately 1 to 2 mg. of carcinogen per pellet). The technical procedures are described by Jull (1951). It was found convenient to distend the bladder at *post mortem* with fixative (Bouin's solution), then to bisect the organ in the saggital plane and to examine the interior with a hand lens or dissecting microscope. By this means tumours could be detected not infrequently. The two halves of the bladder were then embedded on the cut surface. As the work progressed and it was found that tumours did not occur on the suture line of the dome, this portion was cut away before embedding took place (Fig. 5), as cutting the silk sutures tended to tear the sections.

The results of such implantations are shown in Fig. 1 to 4. Experience has shown that epithelial hyperplasia is to be expected even following the implantation of an innocuous pellet such as paraffin wax and therefore this change is not

FIG. 1.—Results of implantation of pellets containing 2-naphthylamine in 8 mice which survived for 20 weeks or longer and did not develop tumours. M = metaplasia.

recorded in the charts. Such hyperplasia tends to reach a maximum between 12 and 20 weeks and to subside in the later stages of the experiment (Fig. 6 and 7). Complications such as leakage of urine, infection of the urinary tract, formation of concretions and blocking of the urethra by the pellet occur. It has not been possible to estimate the mortality from these causes with any degree of accuracy owing to an outbreak of ectromelia, but in spite of this, sufficient mice have survived for 25 weeks or longer to show that the method is practicable and valuable.

2-*Naphthylamine*.—The purified chemical (Bonser, 1943) was used. In 8 mice, one surviving for 22 weeks and 7 surviving from 33 to 39 weeks, no tumours were seen (Fig. 1 and 9). In Mouse 96 (33 weeks) there was generalised epithelial hyperplasia and a localised area of squamous metaplasia high up on the wall of the bladder.

2-*Amino*-1-*naphthol hydrochloride*.—The chemical was synthesised as described by Bonser *et al.* (1951), except in the case of the pellets introduced into Mice 256, 109 and 292, when it was extracted from the urine of a dog fed with 2-naphthylamine (*in vivo* material). Six mice survived from 22 to 28 weeks, and no tumours were seen (Fig. 2). Of 8 mice which survived from 30 to 39 weeks, the epithelium was normal in one (33 weeks); one showed extensive squamous metaplasia at 35 weeks (Mouse 23); in one there was an adenoma at 30 weeks (Mouse 109); and in the remaining 5 mice, carcinomas, 4 of which had progressed to the invasion of muscle, were found (Fig. 12 to 15). In 3 of these mice papillomas were also present and in 2 metaplasias, that in Mouse 76 being of squamous and mucous type (Fig. 11).

*Paraffin wax.*—In this control series standard-sized pellets were made from

FIG. 2.—Results of implantation of pellets containing 2-amino-1-naphthol hydrochloride in 12 mice which survived for 20 weeks or longer. M = metaplasia; P = papilloma or adenoma; C = carcinoma; 80° = pellet made of wax M.P. 80°; + = *in vivo* material.

wax only, except in the case of Mice 85 and 86, when 10 to 15 per cent of ammonium chloride was incorporated in the pellets. Seven mice survived 21 to 30 weeks and 5 mice 30 to 39 weeks (Fig. 3). No tumours or metaplasia were observed (Fig. 8).

3:4:5:6:*Dibenzcarbazole.*—It was suggested by Armstrong and Bonser (1950) that wherever this compound could be brought into contact with the tissues in adequate concentration, carcinogenic action might be anticipated. It was therefore decided to test a sample kindly supplied by Professor E. Boyland of the Chester Beatty Research Institute, Royal Cancer Hospital, London. Eight mice survived for 14 weeks or longer (Fig. 4). The first papilloma appeared at 14 weeks, which was earlier by 16 weeks than any tumour caused by 2-amino-1-naphthol hydrochloride. The first carcinoma occurred at 17 weeks (Fig. 16 and 17). This was earlier by 14 weeks than the first 2-amino-1-naphthol carcinoma and by 8 weeks than the methylcholanthrene-induced tumour described by Jull (1951). Squamous metaplasia was a marked feature in 5 mice.

FIG. 3.—Results of implantation of paraffin wax pellets in 12 mice which survived for 20 weeks or longer and did not develop tumours. 80° = pellet made of wax M.P. 80° ; * = $NH_4Cl$ in pellet.

*Experiment* 2.

*Feeding of mice, rats and rabbits.*

(a) 2-*Naphthylamine* by stomach-tube to IF mice fed on a mixed diet, each mouse weighing on an average 25 g. (Table I) : The maximum tolerated dose was given and treatment was continued until the mice died. The only pathological change attributable to the chemical was extensive but somewhat focal proliferation

TABLE I.—*Administration by Stomach-tube to IF Mice of* 5 *mg.* 2-*Naphthylamine in Arachis Oil* (*twice weekly*) ≡ 400 *mg. per kg. Body-weight per week.*

| | Total mice. | | Weeks of treatment. | | | | Cholangiomas. | | |
|---|---|---|---|---|---|---|---|---|---|
| | F. | M. | 30–39. | 40–49. | 50–59. | 60–72. | F. | M. | %. |
| 2-naphthylamine . | 12 | 13 . | $\frac{0}{3}$ | $\frac{0}{8}$ | $\frac{2}{5}$ | $\frac{8}{9}$ | 5 | 5 | 40·0 |
| Arachis oil . . | 5 | 6 . | 0 | $\frac{0}{7}$ | $\frac{0}{4}$ | 0 | 0 | 0 | 0 |

Numerator = mice with cholangiomas. Denominator = mice dying within the period stated.

of the small intrahepatic bile ducts, associated with mild portal cirrhosis (Fig. 18). This was termed " cholangioma " by Cook, Hewett, Kennaway and Kennaway (1940), denoting a proliferative process which may proceed to neoplasia. This change was not observed in 11 control mice which died before the end of the 59th week nor in breeding mice of the same strain observed for many years.

(b) 2-*Naphthylamine* by stomach-tube to CBA mice fed on a mixed diet, each mouse weighing on an average 30 to 35 g. (Table II) : Again the only pathological changes were in the liver. Half of the experimental mice which survived for 50 weeks developed hepatomas, which were regarded as true tumours. Many grew to a large size, and became pedunculated and then infarcted. Two tumours were histologically malignant. Hepatomas did not occur in a small group of control mice surviving as long as the experimental animals, but benign tumours were present in control breeding mice to the extent of 8·1 per cent (Table II). Although

FIG. 4.—Results of implantation of pellets containing 3:4:5:6-dibenzcarbazole in 8 mice which survived for 14 weeks or longer. M = metaplasia ; P = papilloma or adenoma ; C = carcinoma.

Andervont (1950), has pointed out that variations in incidence of hepatomas must be regarded with caution, this difference would appear to be biologically as well as statistically significant.

TABLE II.—*Administration by Stomach-tube to CBA Mice of* 5 *mg.* 2-*Naphthylamine in Arachis Oil* (*twice weekly*) ≡ 240 *mg. per kg. body-weight per week.*

| | Total mice. | | Weeks of treatment. | | | | | Hepatomas. | | |
|---|---|---|---|---|---|---|---|---|---|---|
| | F. | M. | 30–39. | 40–49. | 50–59. | 60–69. | 70–89. | F. | M. | %. |
| 2-naphthylamine | 14 | 9 | 0 | 0/2 | 1/4 | 2/3 | 10/14 | 7 | 6 | 52·0 |
| Arachis oil | 7 | 7 | 0/2 | 0/5 | 0 | 0 | 1*/7 | 0 | 0 | 0 |
| Breeding | 102 | 46 | 0 | 0/16 | 1/40 | 0/20 | 11/72 | 7 | 5 | 8·1 |

* Single cholangioma.

Numerator = mice with hepatomas. Denominator = mice dying within the period stated.

(c) 2-*Naphthylamine* by incorporation in the diet of CBA mice : The basic diet consisted of casein (20 per cent), starch (50 per cent), arachis oil (10 per cent), yeast (10 per cent), vitamin oil, salts and water to make a paste. The chemical was dissolved in arachis oil and well mixed with the diet. Modifications of the diet were also arranged so that the fat content was high (30 per cent arachis oil), excess of cystine was added (0·5 per cent) or the protein content was reduced (10 per cent casein) and cystine added (0·5 per cent). The amount of the diet eaten on an average by each mouse was weighed, and it was found that only 160 mg. of chemical per kg. of body weight was ingested compared with 240 to 400 mg. by stomach tube (Tables I, II and III). Hepatomas occurred in each group (Table III), the variations in incidence not being significant. Malignant hepatomas were found in 16 mice, one having metastasised to the lungs.

TABLE III.—*Feeding of CBA Mice with 2-Naphthylamine in Adequate Synthetic Diet ≡ approx. 160 mg. per kg. body-weight per week.*

| Diet. | Total mice. | | Weeks of treatment. | | | | | Hepatomas. | | |
|---|---|---|---|---|---|---|---|---|---|---|
| | F. | M. | 40–49. | 50–59. | 60–69. | 70–79. | 80–89. | F. | M. | %. |
| Basic (20% protein) | 12 | 14 | 0 | 0/3 | 1/1 | 2/2 | 8/20 | 5 | 6 | 42·3 |
| High fat (30%) | 12 | 14 | 0 | 1/3 | 0 | 0 | 13*/23 | 9 | 5 | 53·6 |
| High cystine (0·5%) | 15 | 14 | 0/1 | 1/1 | 3/3 | 9/24 | 0 | 4 | 9 | 48·0 |
| Low protein (10%) + High cystine (0·5%) | 15 | 15 | 1/2 | 1/3 | 0 | 8/24 | 1/1 | 7 | 4 | 36·7 |

* Metastasis to the lungs in one mouse.

Numerator = mice with hepatomas. Denominator = mice dying within the period stated.

(d) 2-*Naphthylamine* by incorporation in the diet of rats : Albino rats were supplied by Glaxo Ltd. and were three-quarters grown. The basic diet consisted of casein (10 per cent), starch (80 per cent), arachis oil (5 per cent), yeast (2·5 per cent), vitamin oil and salts. To this was added 2-naphthylamine (0·1 g. per kg.) and water to make a stiff dough. From Table IV it is seen that 4 papillomas of the bladder occurred among 31 rats which survived treatment for 60 weeks or more ; no tumours occurred in the controls. Reduction in the protein content of the diet may have played some part in the appearance of the tumours but the numbers are too small to draw any firm conclusion. Hyperplasia of the bladder epithelium was seen in 6 treated and 5 control rats, and squamous metaplasia in 3 and 5 rats respectively. The nematode *Trichosomoides crassicauda* was seen in the bladder epithelium or free in the lumen of 7 control rats but not in treated animals. No cellular reaction was seen in relation to them, which was the experience of Spitz, Maguigan and Dobriner (1950). The liver changes were of very mild type. Twenty-three experimental rats showed mild portal cirrhosis, compared with 16 control rats. Sixteen experimental rats showed mild bile-duct proliferation compared with 7 control rats. One experimental rat had a well-developed cholangioma and 2 control rats had hepatomas. Thus, while it would seem that there was rather more cirrhosis and bile-duct proliferation in the treated group, these changes were also present in the control group. Two-thirds of the rats in both experimental and control groups showed hyperplasia and hyperkeratosis of the forestomach epithelium ; one-third of both groups had keratinising squamous papillomas of the forestomach.

(e) 2-*Naphthylamine* by spoon to rabbits : Three male and 3 female rabbits were fed on a mixed diet of oats, hay and mangels or green food according to the season. Twice per week 200 mg. of powdered 2-naphthylamine was given orally by means of a spoon. The animals maintained their weight satisfactorily and were killed when it appeared that they would not survive longer (Table V). The epithelial changes in the bladder were of a minor order. In Rabbit 12 there was a tiny transitional-cell benign papilloma at $4\frac{3}{4}$ years (Fig. 19) ; in Rabbit 8 there was epithelial hyperplasia with early downgrowth simulating an adenoma at $5\frac{1}{4}$ years.

---

### EXPLANATION OF PLATES.

FIG. 5.—Bladder of Mouse 82, 2-amino-1-naphthol hydrochloride pellet for 39 weeks. The bladder is viewed from above, after cutting away the dome. The dilated urethral orifice is seen as a dark circle above the centre. Immediately adjacent are 2 small papillomata. To the left and right are projecting tumours. × 4.

FIG. 6.—Bladder wall of Mouse 84, 2-amino-1-naphthol hydrochloride pellet for $4\frac{1}{2}$ weeks, showing simple hyperplasia. × 35.

FIG. 7.—Bladder wall of Mouse 119, paraffin wax pellet for 21 weeks, showing urethral orifice. There is marked epithelial hyperplasia, accentuated by tangential cutting. This is the most advanced change seen in any mouse of this group. × 30.

FIG. 8.—Bladder of Mouse 85, ammonium chloride in paraffin wax pellet for 37 weeks. Wall nearly normal. × 6.

FIG. 9.—Bladder of Mouse 91, 2-naphthylamine pellet for 34 weeks. Wall nearly normal. The dilated ureter is seen at bottom right. × 6.

FIG. 10.—Bladder wall of Mouse 96, 2-naphthylamine pellet for 33 weeks. Localised patch of epithelial hyperplasia close to dilated urethral orifice. × 45.

FIG. 11.—Bladder wall of Mouse 76, 2-amino-1-naphthol hydrochloride (synthetic) pellet for 34 weeks. Squamous metaplasia on left and mucous metaplasia on right. The mucus stained red with muci-carmine. × 60.

FIG. 12.—Bladder wall of Mouse 109, 2-amino-1-naphthol hydrochloride (*in vivo*) pellet for 30 weeks. Adenoma with commencing downgrowth but no invasion of muscle. × 130.

FIG. 13.—Bladder wall of Mouse 81, 2-amino-1-naphthol hydrochloride (synthetic) pellet for 38 weeks. Transitional-cell papilloma, just invading muscle on left. × 60.

FIG. 14.—Bladder wall of Mouse 82, 2-amino-1-naphthol hydrochloride (synthetic) pellet for 39 weeks. Transitional-cell carcinoma with downgrowth in central portion. × 20.

FIG. 15.—Bladder wall of Mouse 24, 2-amino-1-naphthol hydrochloride (synthetic) pellet for 31 weeks. Transitional-cell carcinoma invading the muscular wall of the bladder as far as the epithelium of the dilated ureter. A narrow band of muscle still remains on the right. × 50.

FIG. 16.—Bladder wall of Mouse 126, 3:4:5:6-dibenzcarbazole pellet for 14 weeks. Transitional cell papilloma. × 50.

FIG. 17.—Bladder of Mouse 175, 3:4:5:6-dibenzcarbazole pellet for 17 weeks. The lower half of the bladder is filled with cancer, while the upper part is partially contracted. Some suture material is visible top right. The uterus is seen posterior to the bladder. × 6.

FIG. 18.—IF female mouse, receiving 2-naphthylamine by stomach-tube for 72 weeks. Multiple cholangiomatous areas. × 85.

FIG. 19.—Male Rabbit 12, fed with 2-naphthylamine for $4\frac{3}{4}$ years. Transitional cell papilloma at the dome, with slight hyperplasia of adjacent epithelium. × 30.

5

6

7

8

9

Bonser, Clayson, Jull and Pyrah.

10

11

12

13

14

15

16

17

18

19

Bonser, Clayson, Jull and Pyrah.

TABLE IV.—*Feeding of Rats with 2-Naphthylamine in Synthetic Diets in which the Protein Content was varied (approx. 310 mg. per kg. body-weight per week).*

| Diet + 2-naphthylamine. | Total rats. F. | Total rats. M. | Total rats. Unknown. | Weeks of treatment. 20–29. | 30–39. | 40–49. | 50–59. | 60–69. | 70–79. | 80–89. | Over 90. | Total bladder papillomas. |
|---|---|---|---|---|---|---|---|---|---|---|---|---|
| High protein (20 per cent) | 10 | 6 | 1 | 0/2 | 0/2 | 0/1 | 0/4 | 0/2 | 0/0 | 0/0 | 0/6 | 0/17 |
| Mid protein (10 per cent) | 9 | 5 | 1 | 0/1 | 0/1 | 0/2 | 0/0 | 1/6 | 0/3 | 0/0 | 2/2 | 3/15 |
| Low protein (5 per cent) | 10 | 7 | 1 | 0/0 | 0/0 | 0/5 | 0/1 | 1/8 | 0/0 | 0/0 | 0/4 | 1/18 |
| **Diet alone.** | | | | | | | | | | | | |
| High protein (20 per cent) | 13 | 3 | 1 | 0/2 | 0/1 | 0/0 | 0/0 | 0/3 | 0/1 | 0/3 | 0/7 | 0/17 |
| Mid protein (10 per cent) | 5 | 12 | 0 | 0/1 | 0/7 | 0/3 | 0/1 | 0/2 | 0/0 | 0/0 | 0/3 | 0/17 |
| Low protein (5 per cent) | 7 | 5 | 3 | 0/3 | 0/5 | 0/2 | 0/0 | 0/1 | 0/3 | 0/0 | 0/1 | 0/15 |

Numerator = number of rats with bladder papillomas. Denominator = number of rats dying within the period stated.

TABLE V.—*Oral Administration of 2-Naphthylamine to Rabbits* ≡ 100 *mg. per kg. body-weight per week.*

| No. | Colour. | Sex. | Period of treatment (years). | Approximate total dose of chemical administered (g.) | Changes in bladder. |
|---|---|---|---|---|---|
| 7 | Black | Female | $2\frac{3}{4}$ | 58 | Normal. |
| 9 | ,, | ,, | 4 | 84 | ,, |
| 12 | Chinchilla | Male | $4\frac{3}{4}$ | 100 | Transitional cell papilloma. |
| 11 | ,, | Female | $5\frac{1}{4}$ | 110 | Normal. |
| 10 | ,, | Male | $5\frac{1}{4}$ | 110 | ,, |
| 8 | Wild | ,, | $5\frac{1}{4}$ | 110 | Epithelial hyperplasia with downgrowth. |

*Experiment* 3.

*Subcutaneous injection of mice and rats.*

(a) 2-*Amino*-1-*naphthol hydrochloride* in RIII and stock mice : A fresh suspension of the synthetic chemical was made in arachis oil, and each mouse was injected subcutaneously in the flank once per fortnight with 5 mg. per 100 g. body weight. Great difficulty was experienced in finding the maximum tolerated dose and even this amount caused ulceration of the skin on occasion. Twenty-three mice survived for 40 weeks and 3 subcutaneous spindle-cell sarcomas were observed at the site of injection (Table VI). Other lesions such as hepatoma and leukaemia were observed, but these conditions have also been seen in untreated mice and are not necessarily attributable to the treatment. The bladders were normal.

(b) 2-*Amino*-1-*naphthol hydrochloride* in albino rats, derived from the Glaxo stock : Subcutaneous injections were made as in the mice. Fourteen rats survived to the tumour period and 5 subcutaneous spindle-cell sarcomas were observed at the site of injection (Table VI). All these tumours invaded underlying voluntary muscle and one invaded also the pleural and peritoneal cavities. There were metastases in the lungs from one tumour.

TABLE VI.—*Subcutaneous Injection of Mice and Rats with* 2-*Amino*-1-*naphthol Hydrochloride in Arachis Oil* (5 *mg. per* 100 *gm. body-weight per fortnight.*)

| Animal. | No. M. | No. F. | Weeks of treatment. | Type of lesion. | Survival (weeks). 40–49. | 50–59. | 60–69. | over 70. | Total. incidence. |
|---|---|---|---|---|---|---|---|---|---|
| Stock mice | 6 | 9 | 65 | Subcutaneous sarcoma | 1/0 | 1/3 | 0/5 | 0/7 | 2 |
| | | | | Hepatoma | 0/0 | 1/3 | 3/5 | 0/7 | 4 |
| | | | | Leukaemia | 0/0 | 0/3 | 3/5 | 0/7 | 3 |
| RIII mice | 4 | 4 | 29 | Subcutaneous sarcoma | 0/3 | 1/1 | 0/2 | 0/2 | 1 |
| | | | | Leukaemia | 0/3 | 0/1 | 1/2 | 0/2 | 1 |
| Stock rats | 8 | 6 | Until death | Subcutaneous sarcoma | 1/1 | 3/4 | 1/1 | 0/8* | 5 |

* = 6 rats still alive after 80 weeks of treatment.

Numerator = number of animals showing the lesion. Denominator = number of animals dying within the period stated.

*Experiment* 4.

*Painting of mice.*

2-*Naphthylamine* in IF mice : A saturated solution of the chemical in benzene was painted on the skin between the shoulder-blades once per week. In spite of a satisfactory survival rate (Table VII) no tumours of the skin nor liver occurred.

TABLE VII.—*Painting of Skin of IF Mice with Saturated Solution of 2-Naphthylamine in Benzene.*

| Total mice. | | Weeks of treatment. | | | Tumours of skin. | Tumours of liver. |
|---|---|---|---|---|---|---|
| F. | M. | 40–49. | 50–59. | 60–99. | | |
| 9 | 16 | 7 | 3 | 15 | 0 | 0 |

DISCUSSION.

There is no certain knowledge of the mode of entry of 2-naphthylamine into workers in the chemical industry. Three routes may be involved—ingestion, inhalation, and absorption through the skin. Therefore, after the discovery by Hueper and his colleagues in 1938 (Hueper, 1938 ; Hueper, Wiley and Wolfe, 1938) that bladder cancer could be induced in dogs by feeding the chemical, this method was chosen for testing smaller domestic animals with a view to avoiding the expense and other difficulties inherent in treating dogs. When it had been shown by Bonser, Clayson and Jull (1951) that the metabolite of 2-naphthylamine present in greatest quantity in the urine of the dog after feeding was 2-amino-1-naphthyl sulphuric acid (II), it was necessary to reconsider the mode of exhibition of the various substances in the light of their chemical properties. 2-Naphthylamine base is a relatively stable compound, is oil and benzene-soluble, and can be used for feeding, injection or painting. 2-Amino-1-naphthol hydrochloride is an unstable compound, which is easily oxidised, and is water-soluble. Moreover, from the results of metabolism and biological experiments in various species (Bonser, Clayson and Jull, 1951) it seemed probable that to act as a carcinogen it would require to be present at a certain level of concentration. This contention would be greatly strengthened should the cat prove to be a susceptible animal.

It was for these reasons that the idea of introducing the compound incorporated in a solid vehicle into the lumen of the mouse bladder was explored (Jull, 1951). It was postulated that a high diffusion rate could be expected, that conditions inside the bladder would tend to be anaerobic, and that the exposure of the epithelium to the carcinogen would be almost continuous. In addition, intermediary metabolism by the liver would be eliminated.

The results of Experiment 1 are regarded as sound evidence that 2-amino-1-naphthol hydrochloride is a local carcinogen, whereas 2-naphthylamine is not. This statement is supported by the fact that bladders implanted with paraffin wax remained free from tumours, whereas in bladders implanted with the known carcinogens 20-methylcholanthrene (Jull, 1951) and 3:4:5:6-dibenzcarbazole tumours occurred well within the experimental period of the negative results (Fig. 1 to 4). The induction of tumours by the local action of the metabolite of 2-naphthylamine shows also that there is no factor inherent in the mouse bladder which precludes the development of tumours in this form of carcinogenesis.

Additional evidence for the carcinogenic activity of 2-amino-1-naphthol hydrochloride has been obtained. A few local sarcomas have occurred in mice and rats following subcutaneous injections in oil (Table VI). Those obtained in mice would seem to have significance, as although many subcutaneous injections of the same oil containing oestrogens and other chemicals have been made into mice in this laboratory, no sarcomas have hitherto been observed. The 5 sarcomas in rats are probably of less significance, as the subcutaneous tissues of this species are so singularly reactive to foreign materials of any kind. In 1938 Hueper obtained 3 retothelial sarcomas in mice by intraperitoneal injection of impure 2-amino-1-naphthol in stock mice (Hueper, 1938).

By contrast, no evidence has yet been brought forward to suggest that pure 2-naphthylamine is locally carcinogenic, though our own results of subcutaneous injection in oily solution into mice are not yet ready. The few sarcomas induced by Hackmann (1951) followed the use of the impure technical compound. 2-Naphthylamine does, however, induce distant tumours in mice, in the form of hepatomas in the CBA and cholangiomas in the IF strains. It is suggested that the metabolite is acting locally on the liver cells. Local tumours have not been observed following the feeding of dogs, mice, rats and rabbits. Nor did Rhoads (quoted by Hartwell, 1951), in a large experiment, find tumours elsewhere than in the bladder in the rat. No tumours occurred in mice after painting a benzene solution on the skin (Table VI). In general, aromatic amines are not locally carcinogenic, though there are notable exceptions, such as 2-anthramine (Bielschowsky, 1946), and the amino-stilbenes (Haddow, Harris, Kon and Roe, 1948).

Taking all the above evidence into consideration the case is very strong for accepting 2-amino-1-naphthol or its conjugates as the active carcinogen formed by the metabolism of 2-naphthylamine in man and the dog. It seems worthy of emphasis that such a conclusion would scarcely have been possible without the positive evidence obtained by the direct introduction of the test substances into the mouse bladder. This experiment indicates that it is the metabolite which is carcinogenic and not an impurity present in the 2-naphthylamine, as was suggested many years ago and has recently been re-emphasised by Case and Pearson (1952). The 2-naphthylamine used for the implantations was from the same batch as that used for the feeding and painting experiments. The 2-amino-1-naphthol was synthesised in this laboratory by sodium dithionate reduction of 2-nitroso-1-naphthol, and it seems unlikely that either 2:2-dinaphthylamine or 3:4:5:6-dibenzcarbazole and pyrene, suggested as impurities in 2-naphthylamine by Case and Pearson (1952), would be present in this substance. Moreover, when 2 per cent 20-methylcholanthrene in paraffin wax was implanted into the mouse bladder (Jull, 1951) no tumours were obtained in 4 mice surviving from 21 to 42 weeks. This concentration of carcinogen is very much greater than that suggested for the possible impurities of 2-naphthylamine.

There is no evidence to differentiate between the direct action of 2-amino-1-naphthyl sulphuric acid on the bladder epithelium or its further chemical transformation in the bladder. Such chemical transformation might take the form of hydrolysis to give 2-amino-1-naphthol or the formation of polycyclic or other molecules.

An important observation in both the industrial and experimental diseases is the location of the tumours in the bladder and not in the rest of the urinary tract. The rate of excretion of a dose of 2-naphthylamine administered orally to the dog was shown to reach a peak approximately 4 hours later, (unpublished observation). Thus it is probable that urine containing an effective level of the metabolite passes down the renal tubules, pelvis and ureter for a period of about 4 hours, whereas urine stored in the bladder before, during and after the peak may contain an effective level for a longer period. Some other mechanism may also be involved, as when 2-amino-fluorene was fed to rabbits, ureteric as well as vesical tumours were obtained (Bonser and Green, 1950).

2-Amino-1-naphthol is an ortho-hydroxyamine and the hypothesis is being examined that other carcinogenic aromatic amines are active by virtue of their conversion in the body to compounds of this type. In preliminary experiments, using an impure sample of 1-amino-2-naphthol hydrochloride, supplied by The British Drug Houses Ltd., evidence of carcinogenic activity has been obtained in 5 out of 6 mice by the bladder implantation method (unpublished observation). Walpole, Williams and Roberts (1952) are pursuing similar ideas in the 4-aminodiphenyl series.

The evidence which has been brought forward here to show that a metabolite of 2-naphthylamine is carcinogenic supports the case for a more vigorous pursuit of metabolites from other chemicals which may be taken into the human body. The types of substance which immediately come to mind are the aromatic amines and some of those chemicals which are so liberally used as food-colouring matters at the present time. Further, knowledge about the metabolic changes which take place in other groups of chemicals, while not having immediate practical application as far as industry is concerned, might enable an opinion to be expressed as to whether it would be safe to introduce new types of chemical into industrial or other processes before the damage is actually done.

### SUMMARY.

Preliminary experiments had suggested that 2-amino-1-naphthol hydrochloride was a local carcinogen when introduced directly into the lumen of the mouse bladder (Bonser, Clayson and Jull, 1951). Further experiments have shown that this method is practicable on a larger scale. Metaplasia, papilloma and carcinoma were induced when 2-amino-1-naphthol hydrochloride and 3:4:5:6-dibenzcarbazole were tested, but no tumours were obtained with 2-naphthylamine nor paraffin wax (Fig. 1 to 4).

Additional evidence of the carcinogenic properties of 2-amino-1-naphthol hydrochloride was obtained by the occurrence of a few sarcomas in mice and rats following subcutaneous injection in oil (Table VI).

No local tumours occurred when 2-naphthylamine was administered orally to mice, rats and rabbits. Hepatomas occurred in mice, which suggested that the metabolite was acting locally on the liver cells. A few benign bladder tumours occurred in rats and rabbits at a late date in the experiments (Tables IV and V).

This investigation has been supported by a grant from the Association of British Chemical Manufacturers. We are indebted to Dr. L. H. Stickland and to Dr. E. C. Armstrong for help in carrying out some of the mouse experiments.

### REFERENCES.

ANDERVONT, H. B.—(1950) *J. nat. Cancer Inst.*, **11**, 581.
ARMSTRONG, E. C., AND BONSER, G. M.—(1950) *Brit. J. Cancer*, **4**, 203.
BIELSCHOWSKY, F.—(1946) *Brit. J. exp. Path.*, **27**, 54.
BONSER, G. M.—(1943) *J. Path. Bact.*, **55**, 1.

*Idem*, Clayson, D. B., and Jull, J. W.—(1951) *Lancet*, ii, 286.
*Idem* and Green, H. N.—(1950) *J. Path. Bact.*, **62**, 531.
Case, R. A. M., and Pearson, J. T.—(1952) *Proc. IIe Congrès int. Biochemie*, p. 464.
Clayson, D. B.—(1950) *Biochem. J.*, **47**, xlvi.
Cook, J. W., Hewett, C. L., Kennaway, E. L., and Kennaway, N. M.—(1940) *Amer. J. Cancer*, **40**, 62.
Hackmann, C.—(1951) *Z. Krebsforsch.*, **58**, 56.
Haddow, A., Harris, R. J. C., Kon, G. A. R., and Roe, E. M. F.—(1948) *Philos. Trans. Roy. Soc.*, **241a**, 147.
Hartwell, J. L.—(1951) ‘ Survey of compounds which have been tested for carcinogenic activity ’, U.S. Public Health Service Publication No. 149, p. 102.
Hueper, W. C.—(1938) *Arch. Path.*, **25**, 856.
*Idem* Wiley, F. H., and Wolfe, H. D.—(1938) *J. industr. Hyg.*, **20**, 46.
Jull, J. W.—(1951) *Brit. J. Cancer*, **5**, 328.
Spitz, S., Maguigan, W. H., and Dobriner, K.—(1950) *Cancer*, **3**, 789.
Walpole, A. L., Williams, M. C. H., and Roberts, D. C.—(1952) *Brit. J. industr. Med.*, **9**, 255.